# Rosette‐forming glioneuronal tumor of the fourth ventricle; A case report and review of the literature

**DOI:** 10.1002/ccr3.4355

**Published:** 2021-08-17

**Authors:** Tadeja Verbančič, Janez Ravnik, Rajko Kavalar

**Affiliations:** ^1^ Department of Pathology University Medical Centre Maribor Maribor Slovenia; ^2^ Department of Neurosurgery University Medical Centre Maribor Maribor Slovenia

**Keywords:** frozen section diagnostics, histology, Rosette‐forming glioneuronal tumor, symptoms, treatment

## Abstract

Despite mostly indolent course and favorable postoperative outcome long‐term follow‐up studies are needed to identify the most appropriate therapeutic strategies to minimize surgical morbidity and neurologic injury in patients with RGNT.

## Graphical abstract

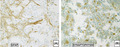

## 1 INTRODUCTION

We present a case of a 34‐year‐old symptomatic male whose radiological imaging and histological examination revealed a rosette‐forming glioneuronal tumor of the fourth ventricle (RGNT). Two months following radical resection of the tumor the patient succumbed due to postoperative complications.

Rosette‐forming glioneuronal tumor (RGNT) is a rare, slow‐growing, mixed neuroglial neoplasm of the central nervous system with a predilection to the fourth ventricle region.^1^ Initially, RGNT was described by Kuchelmeister et al in 1995 as a dysembryoplastic neuroepithelial tumor (DNT) of the cerebellum.^2^ Despite the histological resemblance to DNT, Komory and colleagues established the term RGNT of fourth ventricle and described this tumor as a separate disease entity due to the morphology and biological behavior in 2002.^3^ RGNT was recognized as a new entity of grade 1 brain tumor and was subsequently for the first time included as “RGNT of the fourth ventricle” in the chapter Neuronal and mixed neuronal‐glial tumors of the WHO Classification of Tumours of the Central Nervous System (4th Edition), published by the World Health Organization (WHO) in 2007.^4^

RGNT affects young to middle‐aged adults with a variable female predominance, according to some authors up to 2:1.^1^, ^3^, ^5^, ^6^ No familial association has been described.^7^

A MEDLINE and PubMed searches for terms “rosette,” “forming,” “glioneuronal,” and “tumor” were performed in December 2019 and non‐English articles were excluded. All relevant publications and related articles were scrutinized for RGNT. To our knowledge, about 150 cases have been described in the English literature to present time.^8^, ^9^, ^10^, ^11^, ^12^, ^13^, ^14^

Originally, the location of the RGNT was described as arising exclusively in the fourth ventricle, the vermis, and the cerebellum. However, recent reports show that RGNTs may occur outside the characteristic locations, such as spinal cord, optic chiasm, tectum, pineal gland, third ventricle, cerebellopontine angle, and hypothalamus.^5^, ^15^, ^16^, ^17^, ^18^, ^19^, ^20^, ^21^, ^22^

The spectrum of symptoms is wide and depends on the size and the exact location of the lesion. It includes headache, dizziness, ataxia, dysarthria, blurred vision, vomiting, hemiparesthesia, tinnitus, neck pain, diplopia, nausea, somnolence, hand tremor, anisocoria, and cranial nerve palsy..^8^, ^9^, ^10^, ^21^, ^23^, ^24^ On the other hand, incidentally discovered tumors in asymptomatic patients have also been reported.^8^, ^25^

On computed tomography (CT), RGNT presents as a hypodense mass lesion with/without microcalcifications.^25^

RGNT usually presents on magnetic resonance imaging (MRI) as iso/hypointense on T1‐weighted and hyperintense on T2‐weighted images with limited mass effect and surrounding edema. To a variable amount, it may be enhanced after contrast administration; however, the enhancement is usually nonhomogeneous, rather focal and minimal.^23^, ^25^ The lesions are usually solid or solid/cystic, less often completely cystic. Calcifications have been reported in 21% of cases.^23^ Rarely, satellite lesions can be present.^8^

Microscopically, the tumor displays a typical biphasic morphology; cellular areas with neurocytic rosettes and perivascular pseudo‐rosettes and paucicellular glial areas. Occasionally, Rosenthal fibers of ganglion cell dimension, eosinophilic granular bodies, microcalcifications, and dysmorphic neurons of ganglion cell dimension may be found in glial areas. Necroses and mitoses are usually absent. Immunohistochemically, the glial fibrillary acidic protein (GFAP) is positive in glial component, while cores of neurocytic rosettes and neuropil of perivascular pseudo‐rosettes are immunoreactive to synaptophysin. The proliferation index (Ki‐67) does not precede 1%.^4^, ^6^, ^8^, ^23^, ^24^, ^25^

Clinically and morphologically, RGNT must be differentiated from pilocytic astrocytoma, other low‐grade gliomas with piloid glial component, hemangioblastoma, and glioneuronal tumors arising from the floor of the fourth ventricle or inferior cerebellum.^3^, ^10^, ^26^

Despite the usual favorable postoperative course and prognosis in terms of survival, the location may prevent complete surgical removal, presents a significant risk of neurologic injury and extends postoperative deficits.^6^, ^8^ Surgery with subtotal or gross total resection of tumor mass is the preferred treatment for RGNT and the recurrences are exceptional.^8^, ^20^ Adjuvant radiotherapy and chemotherapy in patients with RGNT are not recommended except in cases with frankly invasive or recurrent tumors.^16^ RGNTs with aggressive behavior in terms of in situ progression/recurrence or dissemination have been reported before.^27^, ^28^, ^29^ Two cases of malignant transformation of a RGNT to glioblastoma were described recently.^13^, ^14^ Only two so far reported RGNTs were associated with IDH‐1 (isocitrate‐dehydrogenase 1) mutation which suggests that IDH‐1 mutation in RGNTs needs further investigation as a biomarker for malignant transformation.^13^, ^30^

## 2 CASE PRESENTATION

A 34‐year‐old Caucasian male with unremarkable medical history was urgently admitted to the Department of Neurosurgery with a 2‐month history of progressively worsening morning headaches accompanied by nausea and vomiting. The pain did not disappear completely after analgesic use and was followed by numbness, tingling sensations and muscle power loss in his upper extremities. Occasionally, the patient also experienced foggy or double vision, vertigo, and clumsy gait. Neurological examination showed both vertical and horizontal nystagmus, accompanied with gait ataxia. Cranial nerve functions were normal.

**FIGURE 1:**
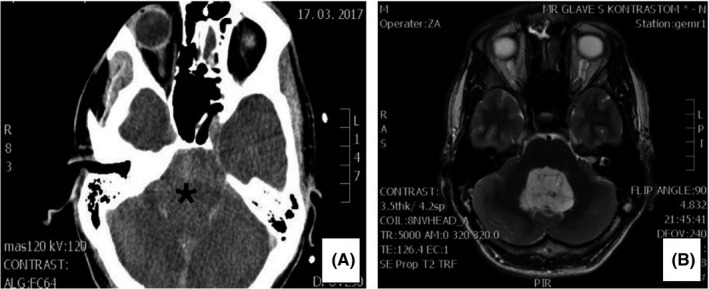
Imaging features of the RGNT. A, CT‐scan with contrast. A mildly hypodense mass lesion (asterisk) in the fourth ventricle measuring 30 × 40 × 40 mm. There was no enhancement after contrast administration. B, MRI, T2‐weighted image. Well‐demarcated, abundant tumor in the fourth ventricle with surrounding edema

Head CT showed a hypodense mass lesion occupying the central part of the posterior cranial fossa, measuring 30 × 40 × 40 mm. The lesion did not demonstrate any contrast enhancement. No calcifications were noticed within the lesion. Surrounding edema of the brain parenchyma and hydrocephalus were identified (Figure 1A).

MRI revealed an abundant, relatively well‐demarcated tumor mass in the fourth ventricle (Figure 1B) causing obstructive hydrocephalus, compression of the cerebellum, and tonsillar herniation.

**FIGURE 2:**
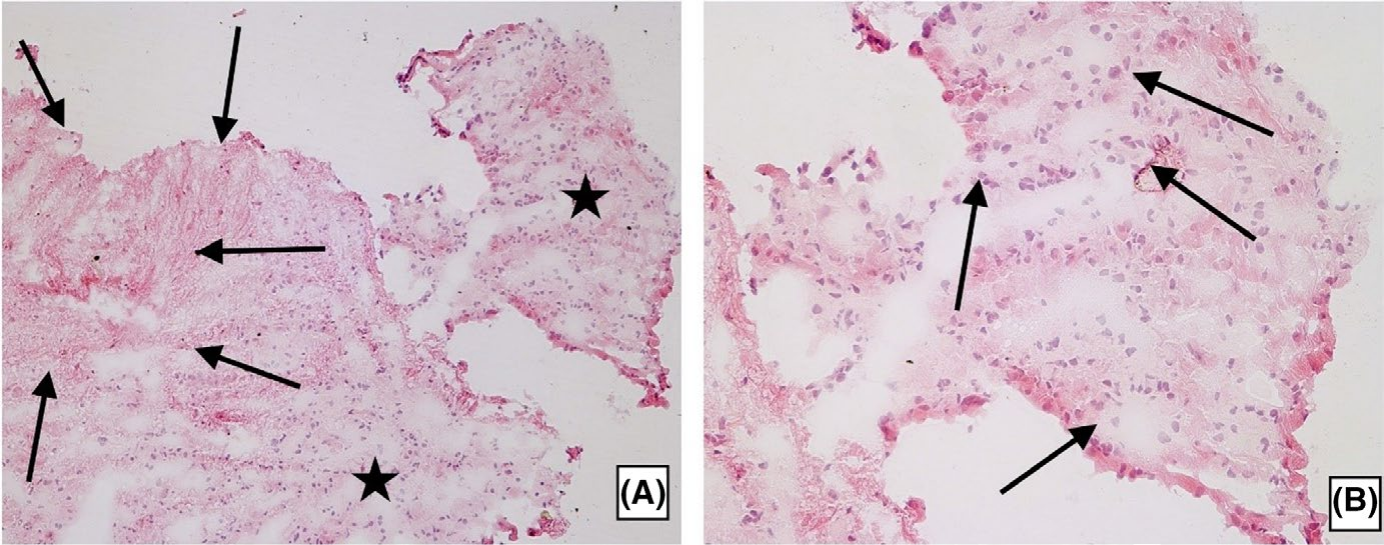
Frozen section of the RGNT. A, Moderately cellular (asterisk) and hypocellular (arrows) areas (H&E × 100). B, Moderately cellular tumor area with implied circle‐like arrangement of the small, bland and uniform nuclei (arrows) (H&E × 200)

The patient underwent a suboccipital midline craniotomy with total resection of the tumor. Several tan and gray fragments of the lesion were submitted for frozen section (FS) and further histopathological examination. On FS examination, the tissue was mostly hypocellular. On one edge of the examined sample, the cellularity was focal slightly pronounced; there were small, uniform, and bland nuclei arranged in a circle‐like fashion. Mitotic activity, necroses, and endothelial proliferation were not present (Figure 2). The report was signed out as a low‐grade glioma.

**FIGURE 3:**
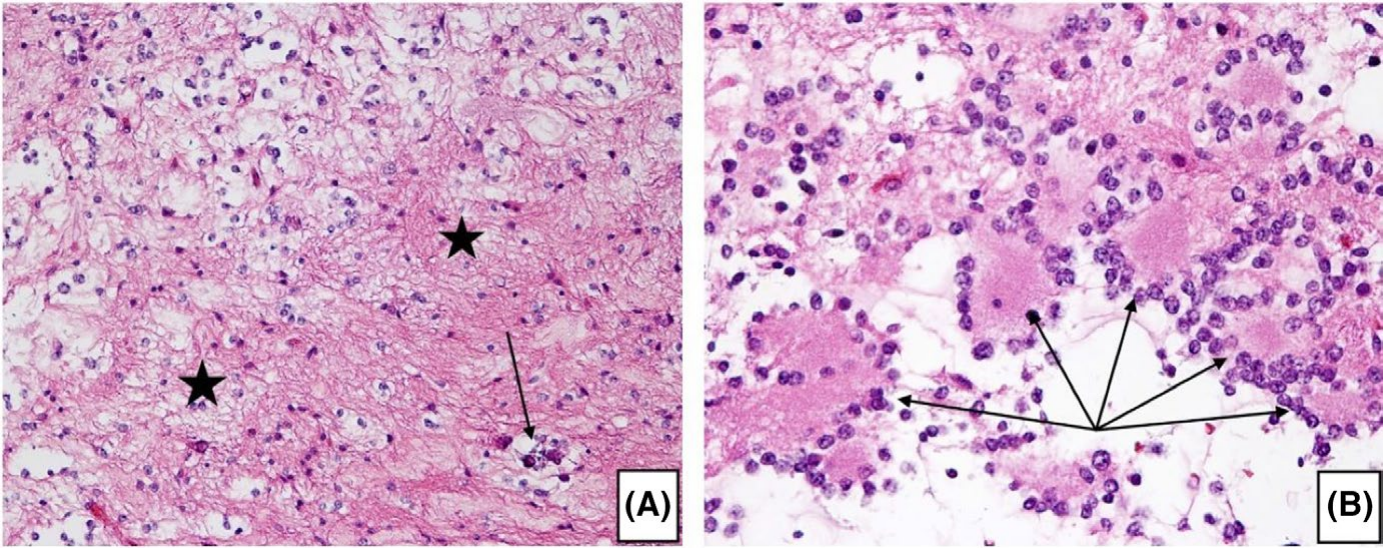
Histopathological features of the RGNT. A, Loose hypocellular astrocytic component (asterisk) with rare microcalcifications (arrow). (H&E, ×200). B, Neurocytes with small, round, regular nuclei with speckled chromatin and scant cytoplasm forming rosettes (arrows) with central eosinophilic neuropil material. (H&E, ×400)

The remaining tumor tissue was formalin‐fixed and embedded in paraffin. The microscopic examination of Hematoxylin & Eosin (H&E) stained sections revealed typical features of a RGNT. The major component of the tumor was represented by neurocytes with small, round, regular nuclei with speckled chromatin and scant cytoplasm forming rosettes with central eosinophilic neuropil material and perivascular pseudorosettes. The counterpart of the neurocytic component was a paucicellular astrocytic component, which consisted of fibrillated spindle and stellate cells with elongated or oval nuclei. Very rare microcalcifications were present and there was no evidence of necrosis, mitotic activity, endothelial proliferation, or nuclear atypia. Ganglion cells and Rosenthal fibers were absent too. Occasional eosinophilic granular bodies were seen (Figure 3).

**FIGURE 4:**
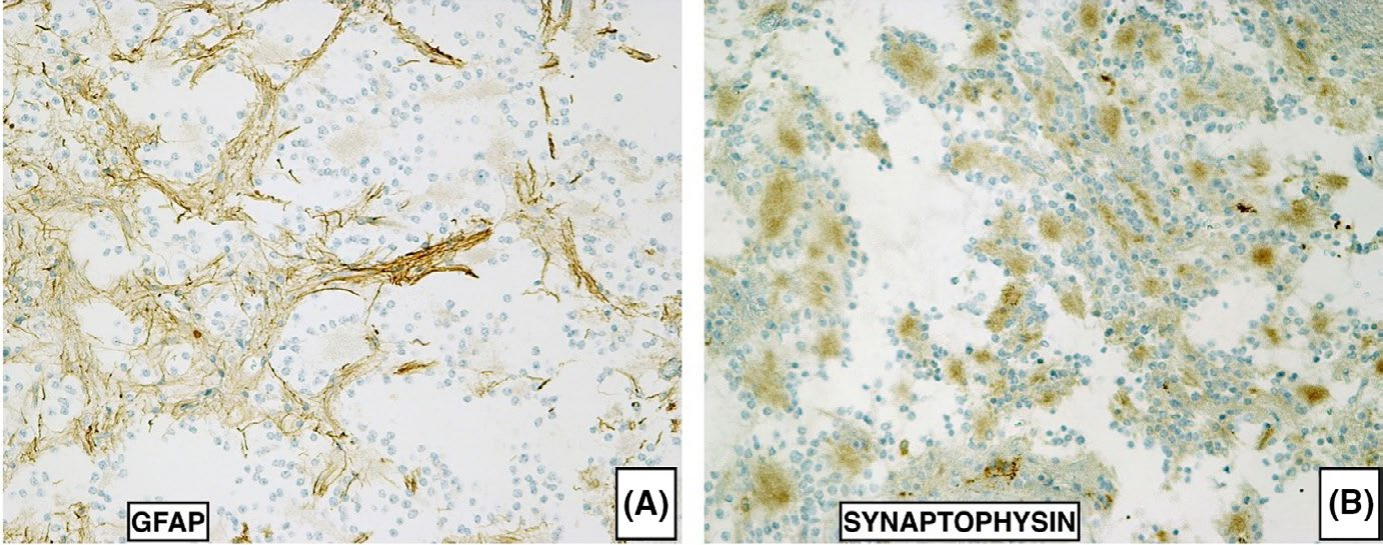
Immunohistochemistry confirms the presence of the A, glial (GFAP—polyclonal, DAKO, ×200) and B, neuronal (Synaptophysin—clone 27G12, Biocare Medical, ×200) differentiation in the tumor

The glial fibrillary acidic protein (GFAP) was immunoreactive in glial component, but absent in rosettes and pseudorosettes. Synaptophysin was positive in the central areas of neurocytic rosettes and in the neuropil of perivascular pseudorosettes, which, on the contrary, did not show any immunoreactivity for the GFAP (Figure 4). The Ki‐67 (MIB‐2) proliferation index was low (<1%).

Immediately after surgery, the patient was awake and cardio‐circulatory stable with Glasgow Coma Scale (GCS) score 15. About eight hours after surgery, he went into cardiac arrest with PEA (pulseless electrical activity) and was successfully resuscitated after more than 10 minutes. However, his pupils were widened and unreactive, he was completely unresponsive (GCS score 3) and hemodynamically unstable with irregular tachyarrhythmia and hypotension. Generalized brain edema and small (12 mm) epidural suboccipital hematoma on the right side were present on a control CT scan and decompressive suboccipital craniectomy was performed. Despite intensive therapy, the patient remained in an unresponsive state with signs of hypoxic encephalopathy on several CT scans. Brain death test results were positive. He died due to pneumonia two months later. An autopsy was performed. Microscopically, the whole brain tissue was avital and no tumor remnants were found.

## 3 DISCUSSION

RGNT is rarely encountered tumor occurring in the fourth ventricle.^2^, ^3^, ^4^ Therefore, our knowledge of this disease is very limited. In our presentation, we have reviewed the literature on the origin, evolution, management, and prognosis of RGNT.

RGNT is a grade 1 tumor entity included in the WHO 2016 Classification of Tumours of the Central Nervous System. According to this classification, RGNT is defined as “a slowly growing neoplasm that affects preferentially young adults, occurs predominantly in the fourth ventricle, can also affect other sites as pineal region, optic chiasm, spinal cord, and septum pellucidum and is composed of two distinct histologic components: one with uniform neurocytes forming rosettes and/or perivascular pseudorosettes, the other being astrocytic in nature and resembling pilocytic astrocytoma”.^1^

The lesion has also been documented in other midline locations within the cerebellum, brain stem, third and lateral ventricles, pineal gland, and hypothalamus.^5^, ^12^, ^15^, ^16^, ^17^, ^18^, ^19^, ^20^, ^21^, ^22^ RGNT, emerging in the fourth ventricle, typically presents with headaches, nausea and vomiting, ataxia and nystagmus due to obstructive hydrocephalus and increased intracranial pressure.^2^, ^4^ Cases of RGNT, located in cerebral hemispheres, just like other supratentorial brain tumors, were associated with epileptic seizures.^31^, ^32^, ^33^

The histogenesis of RGNT is, to date, unclear. The presumed origin of the RGNT is the pluripotent cells of the subependymal plate (periventricular germinal matrix), which have also been suggested as the site of origin of DNTs of the cerebellum.^3^, ^26^, ^34^

It is not known yet whether this finding reflects the normal or aberrant developmental site of precursor or stem cells. The increasing numbers of RGNTs, reported in locations other than the fourth ventricle, cannot exclude the alternate possibility of histologically similar tumors with underlying genetic differences. Although it may resemble a pilocytic astrocytoma, there are no MGMT promoter methylation and no BRAF‐V600E alteration, fusion, as well as mutation, present in either the rosette‐forming or the glial regions of the tumor.^16^, ^35^

The rarity of the lesion and therefore the lack of experience represent a great challenge for the pathologist examining the frozen section (FS) of the RGNT. The location of the lesion, the age and the sex of the patient are helpful data; however, the most important is the biphasic morphology of the lesion. Hypo‐ and hypercellular areas, presence of circle‐like structures made of small, uniform, and bland nuclei, the absence of nuclear atypia, endothelial proliferates, and necroses are the clue to a proper FS diagnosis.^36^, ^37^

Pilocytic astrocytoma, other low‐grade gliomas with piloid glial component, DNT, oligodendroglioma, central neurocytoma, and glioneuronal tumors arising from the floor of the fourth ventricle or inferior cerebellum should be included in the clinical and morphological differential diagnosis of RGNT. The most difficult tumor to differentiate from RGNT is pilocytic astrocytoma, especially if only the astrocytic component is present in the tissue sample. However, pilocytic astrocytoma can be differentiated from RGNT by the absence of rosettes or perivascular pseudorosettes. DNT is typically a supratentorial tumor that presents in younger age group. Oligodendrogliomas with neurolytic differentiation may show neurocytes or even well‐formed rosettes; however, they occur in older age, include oligodendroglial components, and generally arise in frontal lobe. Neurocytoma lacks the biphasic pattern—small round cells forming rosettes are present, yet there is the absence of astrocytic components in the tumor.^1^, ^3^, ^10^, ^11^, ^26^, ^38^

RGNTs are benign brain tumors with the possibility of malignant transformation and IDH‐1 mutation.^13^, ^14^, ^30^ Subtotal or gross total resection of tumor mass is the preferred treatment for RGNT, the recurrences are exceptional and the prognosis in terms of survival is favorable.^6^, ^8^ Unfavorable location of the tumor or disproportional surgical aggressiveness can represent a significant risk of neurologic injury and extended postoperative deficits.^8^, ^11^, ^39^, ^40^ Adjuvant radiotherapy and chemotherapy in patients with RGNT are not recommended except in cases with frankly invasive or recurrent tumors and as a definitive treatment in inoperable cases.^16^, ^40^

Our case elucidates the need for possible changes in surgical techniques to minimize surgical morbidity and neurologic injury in these benign tumors especially before they could progress locally, transform into malignant and become occlusive. Could the use of the stereotactic‐guided Gamma Knife or stereotactic laser‐induced thermotherapy (LITT) be reasonable in RGNT patients as well? Should the prior biopsy help the surgeon in planning the surgical procedure? Do we have to test all RGNTs for IDH‐1 mutation or should this testing be reserved only for cases with frankly malignant transformation? Long‐term follow‐up studies are needed to answer these questions and to identify the most appropriate therapeutic strategies to diminish severe and disabling postoperative neurological deficits in patients with RGNT.

In summary, RGNT is considered a rare, mostly low‐grade tumor entity with distinct radiological, histological, and immunohistochemical features. Although it was initially described as a strictly fourth ventricle lesion, other locations are possible to dictate the clinical symptoms and the choice of treatment. RGNT should always be considered in differential diagnosis with other infratentorial lesions, especially in young adults. Despite mostly indolent course and favorable postoperative outcome, the experience with this tumor remains limited.

## CONFLICT OF INTEREST

The authors declare no conflict of interest.

## AUTHOR CONTRIBUTIONS

TV: involved in conception, design and preparation of the manuscript, acquisition, analysis and interpretation of the gathered data, and critically revised the manuscript.JR: involved in acquisition of the clinical data, performed the surgical procedure, and critically revised the manuscript. RK—the corresponding author— analyzed the histopathological slides (FS, H&E, and IHC) and signed out the final biopsy report, involved in conception, design and preparation of the manuscript, analysis and interpretation of the gathered data, microphotography, and critically revised the manuscript.

## ETHICAL STATEMENT

The authors declare human ethics approval was not needed for this study.

## Data Availability

The data are available from the corresponding author, RK, upon reasonable request.
